# Lactate dehydrogenase is associated with flow-mediated dilation in hypertensive patients

**DOI:** 10.1038/s41598-023-27614-3

**Published:** 2023-01-14

**Authors:** Xiaoqi Cai, Tingjun Wang, Liangdi Xie

**Affiliations:** 1 Department of Geriatrics, Fujian Hypertension Research Institute, The First Affiliated Hospital of Fujian Medical University, 20 Chazhong Road, Fuzhou, 350005 Fujian People’s Republic of China; 2 Department of Geriatrics, National Regional Medical Center, Binhai Campus of the First Affiliated Hospital, Fujian Medical University, Fuzhou, People’s Republic of China; 3 Clinical Research Center for Geriatric Hypertension Disease of Fujian Province, The First Affiliated Hospital of Fujian Medical University, Fuzhou, People’s Republic of China; 4 Branch of National Clinical Research Center for Aging and Medicine, The First Affiliated Hospital of Fujian Medical University, Fujian Province, Fuzhou, People’s Republic of China; 5 Department of General Medicine, The First Affiliated Hospital of Fujian Medical University, 20 Chazhong Road, Fuzhou, 350005 Fujian People’s Republic of China

**Keywords:** Biomarkers, Cardiology, Risk factors

## Abstract

The level of lactate dehydrogenase (LDH) has been proved to be positively associated with albuminuria, which represents glomerular endothelial damage in hypertension (HTN). In this study, the relationship between LDH and endothelial function evaluated by flow-mediated dilation (FMD) was investigated in hypertensives. 1507 subjects (aged 61.2 ± 12.5 years) were enrolled. All hypertensives (n = 1216) were subdivided into 3 groups: LDH1 (lowest tertile of LDH, n = 399), LDH2 (mediate tertile of LDH, n = 409) and LDH3 (highest tertile of LDH, n = 408). Meanwhile, 291 normotensives served as controls. FMD of right anterior tibial artery was assessed by high-resolution color Doppler ultrasound. The level of LDH in hypertensives was significantly higher than normotensives (*p* < 0.001). Whereas, FMD was obviously more blunted in hypertensives (*p* < 0.001). There was an increasing trend of FMD < 8% from control, LDH1, LDH2 to LDH3 group (*χ*^2^ = 36.751, *p* < 0.001). Stepwise multiple liner regression analysis demonstrated an independent correlation between LDH and FMD in hypertensives (β =  − 0.145, *p* < 0.05). After stratified analysis, the relevance persisted in the male, young and middle-aged, hypertensives with grade 2 HTN, duration of HTN < 3 years, metabolic syndrome and those without statin therapy. In conclusion, the level of LDH was inversely correlated with FMD among hypertensives. Those hypertensives with increased LDH need to be scanned for target organ damage, such as microalbuminuria and endothelial dysfunction, and more frequent following up are also recommended.

Lactate dehydrogenase (LDH), a cytoplasmic enzyme widely existing in human body, participates in the process of anaerobic glycolysis^1^. LDH will be released from cytoplasm upon cell injury, and can be detected in the serum^2^. Increased LDH level is demonstrated to be associated with impaired bioavailability of NO, endothelial dysfunction, and end-organ vasculopathy in patients with a syndrome of hemolysis^3^. Moreover, our previous study indicated the level of LDH was positively associated with albuminuria in individuals with hypertension (HTN)^4^. At present, it has been revealed that microalbuminuria not only reflects early renal impairment, but also represents glomerular endothelial damage^5^. As HTN is well recognized to be closely related with endothelial dysfunction^6^, we assume that the association between LDH and albuminuria in hypertensives may result from endothelial injury. Whereas, little is known regarding the association between LDH and endothelial dysfunction in hypertensive patients. It is well-known that flow-mediated dilation (FMD) is the ‘Golden Standard’ for assessing endothelial function, and it is considered to be noninvasive, validated and repeatable, which has been identified as an independent predictor for risk of cardiovascular events^7^. The present study was aimed to explore whether there was an association between LDH and endothelial function assessed by FMD of anterior tibial artery in hypertensives from southern China.

## Methods

### Participants

The present study was conducted in subjects from the database of the research: Target organ damage and related risk factors in hypertensives (registered number: ChiCTR2000039448 (28/10/2020), URL:http://www.chictr.org.cn/index.aspx), a clinical study conducted in Fuzhou city of China. The present study conformed to the ethical standards of the Helsinki Declaration, and the study protocol was authorized by the Branch for Medical Research and Clinical Technology Application, Ethics Committee of the First Affiliated Hospital of Fujian Medical University [reference number: MRCTA, ECFAH of FMU (2020) 306] (Supplementary material). Written informed consent was provided from each participant involved. In accordance with the latest guidelines for the management of HTN^8,9^, HTN referred to systolic blood pressure (SBP) ≥ 140 mmHg and/or diastolic blood pressure (DBP) ≥ 90 mmHg and/or receiving antihypertensive therapy. 160 mmHg > SBP ≥ 140 mmHg and 100 mmHg > DBP ≥ 90 mmHg was recognized as Grade 1 HTN, and SBP ≥ 160 mmHg and/or DBP ≥ 100 mmHg was considered as Grade 2 HTN^9^. Metabolic syndrome (MetS) was diagnosed when a subject met 3 or more of the following standards^8^: 1. Waist circumference (WC) of men ≥ 90 cm and WC of women ≥ 85 cm; 2. SBP/DBP ≥ 130/85 mmHg or having a history of HTN; 3. Triglycerides ≥ 1.7 mmol/L; 4. High-density lipoprotein-cholesterol (HDL-C) < 1.04 mmol/L; 5. Fasting serum glucose ≥ 6.1 mmol/L, 2 h serum glucose ≥ 7.8 mmol/L or already suffering from diabetes mellitus. Exclusion criteria included: 1. secondary hypertension; 2. serum creatinine > 2.5 mg/dL; 3. hepatitis and cirrhosis, level of bilirubin and aminotransferase ≥ 3 times the upper normal limit; 4. acute myocardial infarction and stroke within 3 months; 5. congestive heart failure, ischemic heart disease, severe arrhythmia, valvular heart diseases, hypertrophic cardiomyopathy, restrictive cardiomyopathy; 6. pulmonary hypertension and pulmonary arterial embolism; 7. hyperthyroidism or hypothyroidism; 8. acute or chronic inflammatory diseases; 9. rheumatic autoimmune diseases; 10. chronic consumptive diseases and malignant tumors; 11. skeletal muscle disorders; 12. mild or severe anemia; 13. anterior tibial artery tortuosity or stenosis; 14. women during period of pregnancy and lactation. In this study, 1987 subjects from the inpatients or outpatients of the department of Geriatrics and General Medicine from the First Affiliated Hospital of Fujian Medical University were screened from August 2000 to June 2016. 417 subjects were removed from the sample due to incomplete data, 63 subjects were further excluded according to the exclusion criteria. Eventually, a total of 1507 participants were enrolled and analyzed. The subjects included 1216 hypertensives and 291 age and gender-matched normotensives. According to the level of LDH, all hypertensives patients were further subdivided into 3 groups: LDH1 (lowest tertile of LDH, n = 399), LDH2 (mediate tertile of LDH, n = 409) and LDH3 (highest tertile of LDH, n = 408).

### Clinical data collection

Basic information, general physical examination records, laboratory assays of blood and urine were collected. Subjects were inquiried regarding gender, age, smoking and drinking habits, and medical history on admission. Height and body weight were measured, then the formula as body weight (kg) divided by the square of height (m^2^) was used to calculate body mass index (BMI). WC was measured with a tape along the midway between iliac crest and the lower rib border. Current smoking referred to consuming at least 1 cigarette each day for more than half a year. Drinking referred to alcohol intake > 25 g/d (male) and > 15 g/d (female), alcohol intake = 0.8 (specific gravity of alcohol) × alcoholic beverage (ml) × alcohol concentration. All participants were not allowed to consume coffee or strong tea half an hour before examination. After sitting for more than 5 min in a relaxed position, blood pressure was determined by the automated sphygmomanometer (HEM-7052, Omron, Kyoto, Japan) and heart rate was measured at the same time. The average result of three measurements was then used in the following analysis. All subjects were required to avoid vigorous activities, high-fat and high-salt diet in recent 3 days and blood samples for laboratory assays were taken after 8-h fasting. The levels of total bilirubin (TBil), LDH, aspartate aminotransferase (AST), alanine aminotransferase (ALT), alkaline phosphatase (ALP), gamma glutamyl transpeptidase (GGT), creatinine, uric acid, fasting serum glucose, total cholesterol, triglycerides, low-density lipoprotein-cholesterol (LDL-C) and HDL-C were detected using the autoanalyzer (ADVIA 2400, Siemens, Germany). Analysis was performed to determine white blood cell (WBC) counts by the autoanalyzer (ADVIA 2120, Siemens, Germany). Automatic analyzer (VARIANTTM-II, Bio-Rad, USA) was used to test glycosylated hemoglobin (HbA1c) through high performance liquid chromatography. The first morning urine samples were taken and analyzed within 2 h. Urinary albumin was detected through immunoturbidimetry technique (Roche P800 automatic analyzer, USA) and urinary creatinine concentration was determined by means of colorimetry assay (Boehringer Mannheim/Hitachi 717 system, Germany). Urinary albumin-to-creatinine ratio (UACR) equaled to the ratio of urinary albumin (mg) to urinary creatinine (g).

### Measurement of FMD

All participants were required to fast for over 4 h, and keep away from smoking, taking alcohol, caffeine and vasoactive drugs except for antihypertensive drugs on the day of the FMD measurement. Subjects were demanded to lie in a relaxed supine position in an air-conditioned room with a constant temperature of 25 °C for 15 min. Subsequently, all patients underwent the examination of FMD based on the guidelines for ultrasound detection of FMD modified by our team formerly^10–12^. Briefly, the right anterior tibial artery 10 cm below the transverse popliteal stria was chosen as target vessel, and the longitudinal images of which were recorded by high-resolution 10 MHz linear array transducer (LOGIQ7 system, American GE Company). 3 measurements were conducted for target artery diameter, and the average value was used as the final result. After baseline measurement(D_0_), the inflation (200 mmHg) of a pneumatic cuff (Hokanson) located 5 cm from the proximal end of the fibular head persisted for 5 min, and the diameter (D_1_) was obtained at 60 s post-cuff deflation. Percentage of FMD was calculated as [(D_1_ − D_0_)/D_0_] × 100. FMD < 8% was considered as endothelial dysfunction^13^. Three technicians involved in the measurements received strictly standard training and were blinded from the clinical characteristic of all patients. The intra-observer and inter-observer coefficient of variation for FMD was 1.90% and 6.92% respectively^14,15^.

### Statistical analysis

Continuous variables were tested for normal distribution and homogeneity of variance, the data in accordance with the normal distribution were shown as means ± standard deviation, and continuous variables were presented as median and interquartile distance for abnormal distribution. Categorical variables were displayed as absolute values and percentages. Analysis of variance (ANOVA) was performed for comparisons among groups when the variables were in both normal distribution and equal variance. Otherwise, comparisons among groups were conducted by Wilcoxon signed-rank test. Correlations between FMD and variables were analyzed by Spearman correlation analysis. The independent factors related to FMD were determined by stepwise multiple linear regression analysis. Statistical significance referred to two-side *p* < 0.05. SPSS 20.0 statistical software package (NY, USA) was used to analyze data, and a power analysis of stepwise multiple regression in normotensives and hypertensives was conducted by PASS 11 software.

## Results

### Clinical characteristics of the subjects

**Figure 1:**
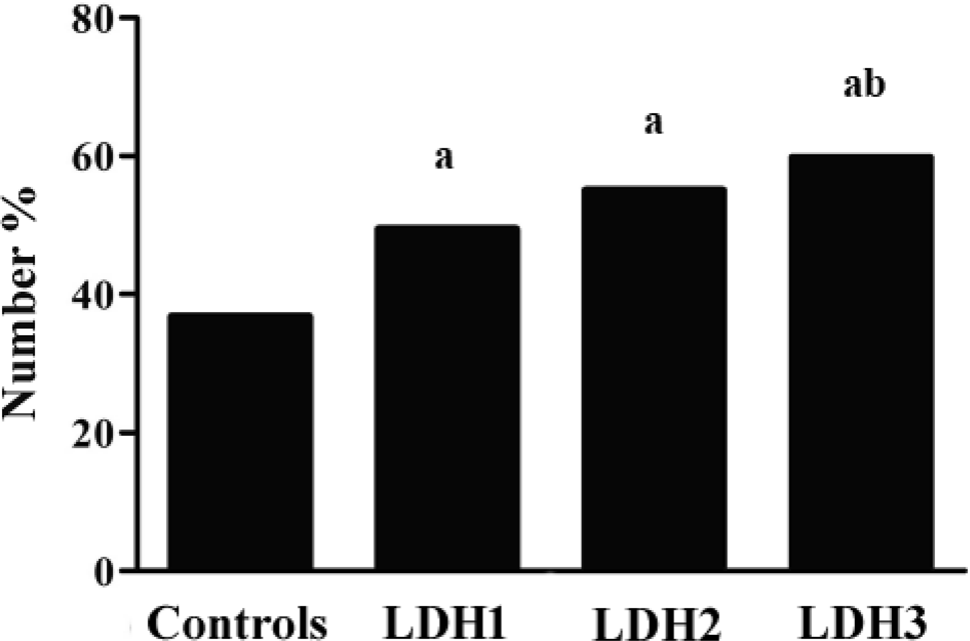
Proportion of endothelial dysfunction in normotensives and hypertensive patients with increasing tertile of LDH level (n = 1507). ^a^*p* < 0.05 versus normotensives, ^b^*p* < 0.05 versus hypertensive patients with lowest tertile of LDH level.

1507 individuals (aged 61.2 ± 12.5 years, 53.8% male) were enrolled in this study. Demographic data of all subjects are summarized in Table 1. Generally, the level of LDH in hypertensives was significantly higher than normotensives [179 (157 − 203)U/L vs 169 (145 − 191)U/L, *p* < 0.001]. FMD was obviously blunted in hypertensives, compared with normotensives [7.41 (4.22 − 10.70)% vs. 9.68 (7.00 − 12.77)%, *p* < 0.001]. In addition, BMI, WC, systolic and diastolic blood pressure, duration of hypertension, ALT, AST, TBIL, GGT, UACR, uric acid, D_0_, MetS rate, the use of angiotensin-converting enzyme inhibitors (ACEIs)/angiotensin receptor blockers (ARBs), β-blockers, diuretics, calcium channel blockers (CCBs), aspirin and statins in patients with HTN were significantly higher than those without HTN. However, total cholesterol, LDL-C, and HDL-C were lower in hypertensives than normotensives. From the lowest to the highest tertile, the median and range of LDH levels in 3 hypertensive subgroups were as following: the lowest tertile was 147.0 (15.0 − 164.0) U/L, second tertile 178.0 (165.0 − 193.0) U/L, and the third quartile 214.5 (194.0 − 655.0) U/L. No apparent difference was found in age, smoking rate, drinking rate, heart rate, WBC, creatinine, fasting serum glucose, triglycerides and HbA1c among the 4 groups. No significant difference was shown in MetS, duration of hypertension, TBil, total cholesterol, LDL-C and use of β-blockers, diuretics, aspirin among 3 subgroups as well. There was an increasing trend of endothelial dysfunction from control, LDH1, LDH2 to LDH3 group (36.8% vs. 49.4% vs. 55.0% vs. 59.8%, *χ*^2=^  = 36.751, *p* < 0.001) (Fig. 1) and among subgroups in hypertensives (χ^2^ = 8.847, *p* = 0.003). FMD in LDH3 group was significantly lower than that of the LDH1 group. In addition, SBP gradually increased with the tirtle of LDH in hypertensives (*p* = 0.027).

**Table 1:** Clinical characteristics of all subjects with and without hypertension.

Variables	Normotensives	LDH1	LDH2	LDH3	*p* value
N	291	399	409	408	
Male, n (%)	149 (51.2%)	238 (59.6%)^a^	226 (55.3%)	198 (48.5%)^b^	0.011
Age (years)	60.6 ± 12.7	61.6 ± 13.3	61.1 ± 11.9	61.1 ± 12.1	0.794
Current smoking [n (%)]	51 (24.1%)	57 (19.5%)	65 (18.6%)	68 (18.8%)	0.169
Drinking [n (%)]	10 (4.8%)	15 (5.1%)	14 (4.0%)	12 (3.3%)	0.264
Duration of hypertension (years)	0 (0–0)	6.0 (2.0 − 12.0)^a^	5.0 (2.0 − 10.10)^a^	7.0 (2.0 − 11.0)^a^	< 0.001
BMI (kg/m^2^)	23.89 ± 3.18	24.27 ± 3.09	24.65 ± 3.16^a^	25.02 ± 3.48^ab^	< 0.001
WC (cm)	86.5 ± 9.6	88.6 ± 9.5^a^	88.99 ± 9.1^a^	89.7 ± 9.4^a^	0.001
Systolic blood pressure ( mmHg)	121.7 ± 10.2	136.4 ± 15.1^a^	138.3 ± 15.4^a^	139.3 ± 16.0^ab^	< 0.001
Diastolic blood pressure ( mmHg)	73.5 ± 7.6	81.2 ± 12.1^a^	82.8 ± 11.1^ab^	82.5 ± 11.9^a^	< 0.001
Heart rate (bp)	72.5 ± 10.3	72.4 ± 9.1	73.0 ± 9.7	73.5 ± 9.7	0.369
WBC (10^9^/L)	5.90 ± 1.49	6.14 ± 1.54	6.17 ± 1.56	6.12 ± 1.65	0.398
TBil (μmol/L)	13.46 ± 5.71	13.98 ± 6.05	14.69 ± 6.24^a^	14.70 ± 5.67^a^	0.015
ALT (U/L)	22.0 (16.0 − 31.0)	22.0 (17.0 − 31.1)	24.0 (18.0–33.0)^ab^	25.0 (18.0 − 35.0)^ab^	0.001
AST (U/L)	23.0 (18.0 − 28.0)	23.0 (19.0 − 27.4)	24.5 (21.0 − 30.0)^ab^	27.0 (23.0 − 33.0)^abc^	< 0.001
LDH (U/L)	169.0 (145.0 − 191.0)	147.0 (132.0 − 156.0)^a^	178.0 (171.0 − 185.0)^ab^	214.5 (203.0–230.8)^abc^	< 0.001
GGT (U/L)	22.0 (15.0 − 34.0)	23.7 (15.0–37.8)	27.0 (19.0–39.5)^ab^	29.0 (20.0 − 45.0)^ab^	< 0.001
ALP (U/L)	71.9 ± 22.3	73.0 ± 24.0	73.6 ± 20.8	79.4 ± 22.2^abc^	0.001
Uric acid (μmol/L)	318.1 ± 90.0	346.7 ± 91.1^a^	357.7 ± 98.0^a^	366.4 ± 98.2^ab^	< 0.001
Creatinine (μmol/L)	69.3 ± 21.1	72.2 ± 20.9	70.5 ± 21.7	69.6 ± 22.1	0.288
UACR ( mg/g)	6.22 (4.83 − 11.07)	9.94 (5.92 − 22.05)	8.19 (5.34–17.88)	14.21 (8.70 − 29.26)^ac^	0.001
Total cholesterol (mmol/L)	5.03 ± 1.08	4.80 ± 1.11^a^	4.84 ± 1.04^a^	4.91 ± 1.13	0.039
Triglyceride (mmol/L)	1.23 (0.90 − 1.73)	1.30 (0.95 − 1.85)	1.35 (0.95 − 1.85)	1.33 (0.91 − 1.85)	0.353
HDL-C (mmol/L)	1.41 ± 0.41	1.28 ± 0.38^a^	1.31 ± 0.36^a^	1.43 ± 0.46^bc^	< 0.001
LDL-C (mmol/L)	3.13 ± 0.93	2.90 ± 1.04^a^	2.97 ± 0.97^a^	2.96 ± 0.99^a^	0.028
Fasting serum glucose (mmol/L)	5.63 ± 1.50	5.78 ± 1.23	5.70 ± 0.96	5.83 ± 1.21	0.164
HbA1c (%)	5.92 ± 1.40	5.88 ± 0.98	5.78 ± 0.78	5.97 ± 1.21	0.271
Metabolic syndrome	42 (14.4%)	144 (36.1%)^a^	146 (35.7%)^a^	169 (41.4%)^a^	< 0.001
ACEI/ARBs [n (%)]	0 (0%)	159 (39.85)^a^	167 (40.8%)^a^	191 (46.8%)^ab^	< 0.001
β-blockers [n (%)]	0 (0%)	89 (22.3%)^a^	91 (22.2%)^a^	97 (23.8%)^a^	< 0.001
Calcium channel blockers [n (%)]	0 (0%)	147 (36.8%)^a^	181 (44.3%)^ab^	180 (44.1%)^ab^	< 0.001
Diuretics [n (%)]	0 (0%)	46 (11.5%)^a^	52 (12.7%)^a^	56 (13.7%)^a^	< 0.001
Aspirin [n (%)]	0 (0%)	28 (7.0%)^a^	35 (8.6%)^a^	36 (8.8%)^a^	< 0.001
Statin [n (%)]	79 (27.1%)	192 (48.1%)^a^	163 (39.9%)^ab^	155 (38.0%)^ab^	< 0.001
D_0_ (cm)	0.27 ± 0.06	0.27 ± 0.07	0.29 ± 0.06^ab^	0.29 ± 0.06^ab^	< 0.001
FMD (%)	9.68 (7.00 − 12.77)	8.00 (4.35 − 11.36)^a^	7.41 (4.23 − 10.5)^a^	6.92 (4.02 − 10.0)^ab^	< 0.001

*BMI* body mass index; *WC* waist circumference; *WBC* white blood cell counts; *TBil* total bilirubin; *ALT* alanine aminotransferase; *AST* aspartate aminotransferase; *LDH* lactate dehydrogenase; *GGT* gamma glutamyl transpeptidase; *ALP* alkaline phosphatase; *UACR* urinary albumin to creatinine ratio; *HDL-C* high-density lipoprotein-cholesterol; *LDL-C* low-density lipoprotein-cholesterol; *HbA1c* glycosylated hemoglobin; *ACEI* Angiotensin-converting enzyme inhibitor; *ARB* angiotensin receptor blocker; *D*_*0*_ baseline diameter of anterior tibial artery; *FMD* flow-mediated dilation.

Data are expressed as means ± SD or median (25th–75th).

^a^*p* < 0.05 versus normotensives, ^b^*p* < 0.05 versus LDH1 group, ^c^*p* < 0.05 versus LDH2 group.

### Correlations between UACR and clinical profile

As shown in Table 2, spearman correlation analysis indicated that LDH slightly associated with FMD in all subjects (*r* =  − 0.124, *p* < 0.001) as well as in hypertensives (r = –0.086, *p* = 0.003). Moreover, age, BMI, WC, duration of hypertension, systolic blood pressure, AST, GGT, ALP, uric acid, creatinine, fasting serum glucose, HbA1c, D_0_ were negatively correlated with FMD in all subjects. Additionally, age, WC, duration of hypertension, AST, uric acid, creatinine, fasting serum glucose, HbA1c, D_0_ were reversely related to FMD in hypertensive patients as well. A significant positive relationship was found between total cholesterol, HDL-C and FMD in all subjects. Also, diastolic blood pressure slightly and positively related to FMD in hypertensives.

**Table 2:** Correlation of clinical profile and FMD in all subjects and hypertensives.

Variables	All sujects (n = 1507)		Hypertensives (n = 1216)	
	r	*p* value	r	*p* value
Age	− 0.234	< 0.001	− 0.260	< 0.001
BMI	− 0.075	0.004	− 0.047	0.105
WC	− 0.116	< 0.001	− 0.088	0.004
Duration of hypertension	− 0.245	< 0.001	− 0.193	< 0.001
Systolic blood pressure	− 0.104	< 0.001	− 0.031	0.288
Diastolic blood pressure	0.013	0.605	0.092	0.001
Heart rate	0.009	0.740	− 0.003	0.913
WBC	0.010	0.766	0.039	0.300
TBil	− 0.011	0.667	0.011	0.710
ALT	− 0.033	0.202	0.007	0.798
AST	− 0.090	< 0.001	− 0.066	0.022
LDH	− 0.124	< 0.001	− 0.086	0.003
GGT	− 0.071	0.006	− 0.030	0.296
ALP	− 0.076	0.026	− 0.061	0.104
Uric acid	− 0.084	0.002	− 0.061	0.035
Creatinine	− 0.086	0.001	− 0.092	0.002
UACR	− 0.063	0.312	− 0.033	0.622
Total cholesterol	0.062	0.016	0.045	0.115
Triglyceride	− 0.003	0.918	0.017	0.546
HDL-C	0.060	0.019	0.023	0.414
LDL-C	0.022	0.391	0.017	0.566
Fasting serum glucose	− 0.112	< 0.001	− 0.115	< 0.001
HbA1c	− 0.094	0.006	− 0.077	0.040
D_0_	− 0.202	< 0.001	− 0.176	< 0.001

*FMD* flow-mediated dilation; *BMI* body mass index; *WC* waist circumference; *WBC* white blood cell counts; *TBil* total bilirubin; *ALT* alanine aminotransferase; *AST* aspartate aminotransferase; *LDH* lactate dehydrogenase; *GGT* gamma glutamyl transpeptidase; *ALP* alkaline phosphatase; *UACR* urinary albumin to creatinine ratio; *HDL-C* high-density lipoprotein-cholesterol; *LDL-C* low-density lipoprotein-cholesterol; *HbA1c* glycosylated hemoglobin; *D*_*0*_ baseline diameter of anterior tibial artery.

### Stepwise multivariate linear regression analysis of FMD

To correct confounding effects of variables on FMD, stepwise multiple linear regression analysis with all variables from Table 1 was conducted to evaluate the independent influencing factors for FMD. As indicated in Table 3, LDH was slightly but independently correlated with FMD in all subjects (β =  − 0.162, *p* < 0.05) and hypertensives (β =  − 0.145, *p* < 0.05), but not in patients with normal blood pressure. A sample size of 291 in normotensives achieved 100% power to detect an R-Squared of 0.499 attributed to 33 independent variable(s) using an *F*-Test with a significance level (alpha) of 0.05. The sample size of 1216 in hypertensives achieved 100% power to detect an R-Squared of 0.247 attributed to 33 independent variable(s) using an F-Test with a significance level (alpha) of 0.05. After further stratified analysis in hypertensives (Table 4), the correlation persisted in the male (β =  − 0.280, *p* < 0.01), young and middle-aged (age < 65 years, β =  − 0.237, *p* < 0.01), hypertensives with grade 2 HTN (β =  − 0.293, *p* < 0.05), duration of HTN < 3 years (β =  − 0.326, *p* < 0.05), Mets (β =  − 0.232, *p* < 0.05) and those without statin therapy (β =  − 0.216, *p* < 0.05).

**Table 3:** Stepwise multiple linear regression analysis of FMD before and after stratification of hypertension.

	R^2^	N	Variables	β	t	*p* value
All subjects	0.255	1507	Age	− 0.396	− 5.584	0.000
			Diuretic	0.191	2.872	0.005
			D_0_	− 0.186	− 2.871	0.005
			Statin	0.189	2.638	0.009
			LDH	− 0.162	− 2.492	0.014
Normotensives	0.499	291	Age	− 0.820	− 4.203	0.001
			HbA1c	0.513	2.632	0.017
Hypertensives	0.247	1216	Age	− 0.378	− 5.026	0.000
			Statin	0.209	2.745	0.007
			Diuretic	0.200	2.806	0.006
			D_0_	− 0.178	− 2.565	0.011
			LDH	− 0.145	− 2.082	0.039

*FMD* flow-mediated dilation; *D*_*0*_ baseline diameter of anterior tibial artery; *LDH* lactate dehydrogenase; *HbA1c* glycosylated hemoglobin Diuretic: Yes = 1, No = 0, Statin: Yes = 1, No = 0.

**Table 4:** Stepwise multiple linear regression analysis of FMD in hypertensives after stratification of age, gender, level of HTN, duration of HTN, MetS and statin therapy.

	R^2^	N	Variables	β	t	*p* value
Age < 65 years	0.189	701	Diuretic	0.245	2.796	0.006
			D_0_	− 0.210	− 2.419	0.017
			LDH	− 0.237	− 2.681	0.008
			GGT	0.226	2.572	0.011
Age ≥ 65 years	0.181	515	Diuretic	0.337	2.577	0.001
			WC	− 0.275	− 2.100	0.041
Male	0.288	662	Diuretic	0.309	3.123	0.003
			Age	− 0.195	− 2.049	0.044
			GGT	0.284	2.830	0.006
			LDH	− 0.280	− 2.803	0.006
			Glucose	− 0.253	− 2.496	0.015
Female	0.538	554	Age	− 0.571	− 6.871	< 0.001
			WC	− 0.365	− 4.206	< 0.001
			ALP	− 0.286	− 3.473	0.001
			Triglycerides	0.270	3.124	0.003
			CCBs	0.266	3.175	0.002
SBP/DBP < 160/100 mmHg	0.224	1021	Diuretic	0.259	3.185	0.002
			Age	− 0.322	− 3.751	< 0.001
			Statin	0.243	2.829	0.005
			D_0_	− 0.190	− 2.356	0.020
SBP/DBP ≥ 160/100 mmHg	0.515	195	Age	− 0.650	− 5.516	< 0.001
			LDH	− 0.293	− 2.485	0.018
Duration of HTN < 3 years	0.449	394	BMI	− 0.397	− 3.634	0.001
			LDH	− 0.326	− 3.067	0.003
			β − blockers	0.273	2.610	0.012
			gender	0.216	2.021	0.049
Duration of HTN ≥ 3 years	0.334	822	Age	− 0.310	− 3.415	0.001
			Diuretic	0.210	2.419	0.017
			GGT	0.256	2.965	0.004
			HbA1c	− 0.278	− 3.309	0.002
			Statin	0.198	2.259	0.026
			D_0_	− 0.168	− 2.027	0.045
Non − MetS	0.376	757	GGT	0.530	5.690	0.000
			HbA1c	− 0.283	− 3.369	0.001
			Gender	0.188	2.017	0.047
			Diuretic	0.245	2.888	0.005
			WC	− 0.181	− 2.074	0.041
MetS	0.432	459	Age	− 0.561	− 4.917	< 0.001
			LDH	− 0.232	− 2.277	0.026
			D_0_	− 0.371	− 3.503	0.001
			Statin	0.276	2.317	0.024
			Drinking	0.218	2.141	0.036
Without statin therapy	0.355	706	Age	− 0.512	− 6.509	< 0.001
			Smoking	− 0.246	− 3.130	0.002
			LDH	− 0.216	− 2.756	0.007
With statin therapy	0.242	510	Age	− 0.317	− 2.607	0.012
			GGT	0.341	2.801	0.007

*FMD* flow-mediated dilation; *HTN* hypertension; *MetS* metabolic syndrome; *D*_*0*_ baseline diameter of anterior tibial artery; *LDH* lactate dehydrogenase; *GGT* gamma glutamyl transpeptidase; *WC* waist circumference; *ALP* alkaline phosphatase; *BMI* body mass index; *HbA1c* glycosylated hemoglobin; *CCBs* Calcium channel blockers (Yes = 1, No = 0), Diuretic: Yes = 1, No = 0, Statin: Yes = 1, No = 0, β-blockers: Yes = 1, No = 0, Gender: male = 1,female = 2, Drinking: Yes = 1, No = 0,Smoking: Yes = 1, No = 0.

## Discussion

In this study, we performed a retrospective analysis using the data from Fuzhou study and found that gradually decreased endothelial function was observed along with the increase of LDH level in hypertensives. After stratified analysis in hypertensives, it was shown that LDH independently associated with FMD in the male, young and middle-aged, hypertensives with grade 2 HTN, duration of HTN < 3 years, Mets and those without statin therapy. Although this association was mild, it was demonstrated to be independent of multiple previous reported cardiovascular risk factors. To our knowledge, the present study is the first to investigate the relationship between LDH and FMD of anterior tibial artery in patients with HTN.

HTN is well-known to be closely related with endothelial dysfunction, which is recognized as a predictor of long-term atherosclerotic disease as well as cardiovascular events^16^. As an enzyme widely exists in almost every cell of the body, LDH converts pyruvate to lactate under conditions of oxygen insufficiency^17^. Elevated level of LDH is not only a sign of hypoxia, but also indicates oxidative stress and inflammation^18^, which are important pathophysiological mechanisms for endothelial dysfunction in hypertension. Elevation of LDH is a sensitive indicator of increased cell membrane permeability^19^ and cell injury as well. The process of atherosclerosis is accompanied with increased cell membrane permeability and endothelial cell impairment, which may result in the release of LDH into serum. Furthermore, atherosclerosis is a state with vascular accumulation of lipid plaques, and endothelial injury induced by oxidized low-density lipoprotein (ox-LDL) is considered as the major contributing factor. On the first hand, previous studies^20,21^ showed ox-LDL exposure reduced human umbilical vein endothelial cells (HUVECs) viability and increased LDH release. On the other hand, cholesterol crystal induced endothelial cell inflammation and pyroptosis play a vital role in the development of atherosclerosis as well. A previous experiment^22^ in HUVECs demonstrated that exogenous cholesterol crystal stimulation could lead to elevation of LDH release and cell viability decrease. Ji-Hyun Kim et al.^23^ found that activation of primary rat vascular smooth muscle cells (VSMCs) increased their glycolytic activity, proliferation, migration and expression of LDH-A, which was a subunit of the LDH isoenzyme. Furthermore, knockdown of LDH-A and inhibition of LDH-A both significantly inhibited proliferation and migration of VSMCs. Taken together, this study suggests that LDH-A is involved in the vessel lumen constriction which may directly affect vasodilation. These postulations mentioned above may explain the relationship between LDH and FMD in hypertensives and the possibility that LDH involves in the process of atherosclerosis. However, this relation could not be explained as cause and effect, and LDH may not be used as a direct predictor of atherosclerosis clinically at this stage. In addition, LDH may be influenced by vascular lesion as well as injury of other organs, which may be the reason for the relatively weak correlation of LDH and FMD.

In this study, the correlation between LDH and FMD differed in subgroups based on gender. It was in accordance with our former study, indicating a positive relationship between LDH and intima media thickness of carotid artery only preserved in the male patients with HTN^24^. The diverse results between the gender might be explained by the positive influence of estrogen for female on endothelial function. Additionally, WC, but not LDH, was associated with FMD in the elderly hypertensives, which was in consistent with our recent studies^25,26^ as well. Senior hypertensives and those with a longer history of hypertensive duration are frequently accompanied with multiple cardiovascular risk factors and clinical complications^27^, of whom the FMD may be affected by various factors. Therefore, the interaction and relationship of LDH and FMD may be less notable in these subjects. As the existing research data are limited, further studies are required to investigate the relevant mechanism. As early as 17 years ago, the Framingham Heart Study has revealed that the severity of HTN was positively related to the level of endothelial dysfunction^28^. Jurva JW et al.^29^ also reported the increase of blood pressure level by weight lifting could cause transient decline of FMD, indicating higher shear stress may directly impair endothelial cells. Hence, LDH correlated more closely with FMD in patients with grade 2 HTN.

At present, about 33.9% of adults in China suffer from MetS^30^ which contributes to the mortality worldwide^31^. Nonalcoholic fatty liver disease (NAFLD) is well known to usually occur in the context of MS^32^, and NAFLD is frequently accompanied with increase hepatic enzymes activities, including LDH^33^. This maybe one of the reasons that LDH correlated more closely with FMD in MetS. Insulin resistance is well-recognized as one of the mechanisms for pathophysiology of MetS. Other mechanisms include oxidative stress and low-grade chronic inflammation^31^. Activation of ox-LDL and reactive oxygen species in MetS results in cellular damage and apoptosis^34^, following by release of LDH and endothelial dysfunction. In addition, the statins are well known to have pleiotropic effects on the cardiovascular system, as upregulating the endothelial nitric oxide synthase, enhancing the mobilization of endothelial progenitor cells, reducing the production of reactive oxygen species and proinflammatory cytokines^35^. Therefore, the relevance of LDH and FMD presented less significantly in hypertensives with statin therapy, compared with those did not receive statin therapy.

Biomarkers of endothelial dysfunction include asymmetrical dimethylarginine (ADMA), ox-LDL, endothelial microparticles (EMPs), endothelial progenitor cells (EPCs), and endothelial glycocalyx^36^. Whereas, FMD, quantitative coronary angiography, positron emission tomography (PET), peripheral arterial tonometry (PAT)^37^, invasive measurement of forearm blood flow (FBF) by venous occlusion plethysmography (VOP), and laser Doppler flowmetry (LDF) endothelial microparticles are vascular methods used in the evaluation of endothelial dysfunction^36^. Of all the above, FMD is considered the most reproducible, predictive, inexpensive, low-risk, and non-invasive method of endothelial function evaluation^38^. In recent decades, measurement of flow-mediated vasodilation (FMD) has been widely used for assessing endothelial function in mankind^6,39^. The brachial artery was used as the target vessel of FMD measurement in most studies. According to ‘Expert consensus and evidence-based recommendations for the assessment of flow-mediated dilation in humans^40^’, FMD is typically examined in, but not limited to, the brachial artery (diameter 3–5 mm). Smaller (e.g. radial artery: diameter 1.5–3 mm) or larger sized arteries (e.g.superficial femoral artery: diameter 5–7 mm, popliteal artery: diameter 4–6 mm) were acceptable as well. The baseline diameter of anterior tibial artery of all subjects was 2.81 ± 0.64 mm in the present study. It is well-known that artery blood flow resistance is inversely proportional to the radius of the vessel raised to the fourth power. Thus, slight changes in smaller vessel can more significantly affect vascular resistance. Compared with brachial artery, anterior tibial artery is theoretically more representative and sensitive as the resistance vessel in hypertension. As early as 20 years ago, we used both brachial artery and anterior tibial artery as target vessels to assess FMD in the same subject, and more significant vasodilation of anterior tibial artery was shown in our previous study^41^. In the past decades, we have dedicated to the study of FMD. Several articles^12,14,24,25,41–43^ have been published using anterior tibial artery as target vessel, demonstrating the methodological approach was stable and reproducible.

Some limitations should be stated in the present study. Firstly, this was a cross-sectional study, and no causal relevance can be inferred from the relatively small correlation coefficient of LDH and FMD. In addition, LDH is a sensitive but nonspecific biomarker, it will be better to detect LDH isoenzyme to help identifying the origin of increased LDH. Unfortunately, we do not run this test in our hospital. Secondly, the sample size of the normotensives was relatively small. Therefore, a prospective study with larger sample size is required to verify the results. Thirdly, the mechanisms underlying endothelial dysfunction are multifactorial, the analysis may not be comprehensive enough in this study, as ADMA, ox-LDL, EPCs and other markers related to endothelial function were not measured. In addition, those who received the medication were not excluded, it was possible that the lower levels of LDL-C and total cholesterol in hypertensives resulted from the more frequent use of statins in the subgroup. Nevertheless, the use of antihypertensive drugs, aspirin and statins were taken into consideration in the multiple liner regression to eliminate the influence of pharmacological therapies.

## Conclusion

In conclusion, the level of LDH was correlated with FMD in hypertensive patients. This association was independent of gender, age, blood pressure and multiple risk factors. The present study indicates hypertensives with elevated LDH may have a higher risk of endothelial dysfunction assessed by FMD, which is a potential therapeutic target for hypertensives. However, FMD assessment may not be available in some primary hospitals. Clinically, it is notable for physicians, protective, intensive treatment and frequent following up are needed for hypertensives with elevated LDH. Still, further studies are needed to clarify the mechanism of the association between LDH and FMD.

## Supplementary Information

Supplementary Information.

## Data Availability

The data that support the findings of this study are available from Liangdi Xie, upon reasonable request.
