# Supplementary material for: Lactate dehydrogenase is associated with flow-mediated dilation in hypertensive patients
Source: Sci Rep. 2023 Jan 14;13:768. doi: 10.1038/s41598-023-27614-3 (PMC9840603; doi:10.1038/s41598-023-27614-3)
Supplement: Supplementary file 1 — Supplementary Information. [file 41598_2023_27614_MOESM1_ESM.pdf]

福建医科大学附属第一医院医学伦理委员会医学研究与临床技术应用分会审查批件

Ethics Review Form for Branch for Medical Research and Clinical Technology Application, Ethics Committee of First Affiliated Hospital of Fujian Medical University

批 件 号： 闽医大附一伦理医研[2020]306 号

Approval No. :MRCTA,ECFAH of FMU [2020]306

**项目名称 Protocol Title**	**高血压靶器官损害及其危险因素——福州研究**		
**项目来源 Protocol Source**	**自选课题**		
**主要研究者 Principal Investigator**	**谢良地 Liangdi Xie**	**职 称 Professional Title**	**主任医师 Chief physician**
**伦理审查方式 Mode of Review**	□会议审查（Meeting Review） □√快速审查（Quick Review）		
**审查类别 Type of Review**	□√初次审查 （Initial Review） □修正案审查 （Review on Amendments）<br>□跟踪审查（Tracing Review） □违背方案审查（Review on Contrary to Protocol）<br>□严重不良事件审查（Review on Serious Adverse Events）<br>□暂停/终止研究审查（Suspending/Terminating Review on Researches）<br>□结题审查（Review on End of Research） □复审（Check Review）		
**送审材料 Documents for Reviewing**	1、伦理审查申请表 Application Form<br>2、研究方案（V1.0 2020 年 8 月 24 日）<br>3、知情同意书 Informed Consent Form（V1.0 2020 年 8 月 24 日）		
**审查意见 Comments**	本审查委员会声明：遵从 ICH-GCP、中国 GCP、中国相关法规及指南的要求组成和开展工作，其审查和工作过程不受伦理委员会以外的任何组织及个人的影响。根据国家《药物临床试验质量管理规范》、《赫尔辛基宣言》及《涉及人的生物医学研究伦理审查办法》等伦理原则，对本项目进行伦理快速审查，意见如下：本项目符合伦理要求，同意本项目在我院开展。<br>The Institutional Review Board(IRB) declares that: In accordance with the requirements of ICH-GCP, China GCP and relevant laws and regulations or guidelines in China, the review and other working processes are free from any influence of any organization or individuals other than this Ethics Committee. According to the national		

	ethical principles of *Good Clinical Practice*, *Declaration of Helsinki* and *Ethical Review Methods for Biomedical Research on Human Beings*, a quick ethical review of the project was conducted. The opinions are as follows: The project meets the requirements ， and is approved to be carried out in our hospital.<br><br>主任委员签字：<br>Signature of the chairman of committee:<br>日期 Date（yyyy/mm/dd）：2020 年 9 月 15 日<br>福建医科大学附属第一医院医学伦理委员会<br>医学研究与临床技术应用分会（盖章）<br>Branch for Medical Research and Clinical Technology Application, Ethics Committee of the First Affiliated Hospital of Fujian Medical University (Seal)		
**伦理委员会地址**<br>**Ethics Committee Address**	福建省福州市茶中路 20 号<br>No.20,Chazhong Road,Fuzhou,Fujian Province	**联系电话**<br>**Telephone**	0591-87981028
